# Internet-based cognitive rehabilitation for WORking Cancer survivors (i-WORC): study protocol of a randomized controlled trial

**DOI:** 10.1186/s13063-020-04570-1

**Published:** 2020-07-20

**Authors:** Kete M. Klaver, Saskia F. A. Duijts, Chantal A. V. Geusgens, Maureen J. B. Aarts, Rudolf W. H. M. Ponds, Allard J. van der Beek, Sanne B. Schagen

**Affiliations:** 1 Division of Psychosocial Research and Epidemiology, Netherlands Cancer Institute, Plesmanlaan 121, 1066 CX Amsterdam, The Netherlands; 2 Department of Public and Occupational Health, Amsterdam Public Health research institute, Amsterdam UMC, Vrije Universiteit Amsterdam, Amsterdam, The Netherlands; 3 Netherlands Comprehensive Cancer Organisation (IKNL), Utrecht, The Netherlands; 4 Department of Medical Psychology, Zuyderland MC, Sittard, The Netherlands; 5 Department of Medical Oncology, Maastricht University MC, Maastricht, The Netherlands; 6 Department of Medical Psychology/School of Mental Health and Neurosciences (MHeNS), Maastricht University MC, Maastricht, The Netherlands; 7 Brain and Cognition group, University of Amsterdam, Amsterdam, The Netherlands

**Keywords:** Cancer-related cognitive impairment, Cognitive rehabilitation, Employment, Internet-based, Randomized controlled trial, Cost-effectiveness

## Abstract

**Background:**

Cognitive problems are common in non-central nervous system cancer survivors. These problems are perceived as an important contributor to decline in work performance and work ability. Various interventions for cognitive problems have been proposed, but effectiveness regarding work-related outcomes has not yet been established. Effective treatment options to alleviate the adverse influence of cognitive problems on work performance are needed for working cancer survivors. In this paper, we will describe the design of a randomized, controlled, multicenter trial that evaluates the (cost-)effectiveness of an Internet-based cognitive rehabilitation program for occupationally active cancer survivors confronted with cognitive problems.

**Methods/ design:**

A three-armed randomized controlled trial will be conducted, including two intervention groups (i.e., basic and extensive cognitive rehabilitation program) and one waitlist control group. In total, 261 cancer survivors (18–65 years) who have returned to work and who experience cognitive problems will be recruited. Patients with and without cognitive impairment as established in a neuropsychological assessment will be eligible; stratification will take place based on the presence of this cognitive impairment. The extensive intervention arm will contain a comprehensive training program (including psycho-education, fatigue management, and cognitive strategy training) with individual guidance (blended intervention). The basic intervention arm will contain a brief cognitive training program (including psycho-education and fatigue management) without individual guidance. The primary outcome will be accomplishment of an individually defined work-related treatment goal. Secondary outcomes include, among others, subjective cognitive functioning, work functioning, and quality of life. Primary and secondary outcomes will be measured at baseline (T0) and at 12 weeks (T1) and 26 weeks (T2) post-randomization.

**Discussion:**

About 40–50% of the cancer patients worldwide are of working age at time of diagnosis. Many of the occupationally active cancer survivors experience cognitive problems. Both from an individual and a societal perspective, it is important to sustain cancer survivors’ employability. An effective treatment to alleviate the impact of cognitive decline and to improve work ability might help cancer survivors to sustain employability.

**Trial registration:**

ClinicalTrials.gov NCT03900806. Registered on 03 April 2019 (current status: ongoing).

## Background

Non-central nervous system (CNS) cancer and its treatment can be associated with cognitive problems [1–4]. Up to 78% of non-CNS cancer patients have reported to experience cognitive problems in the active treatment period. While previous studies have shown that cognitive problems improve after completion of therapy [5, 6], a substantial subgroup (approximately 30–40%) of patients is confronted with cognitive problems that can last for months or even up to 10 years post-therapy [1, 5–8]. Although cognitive problems in non-CNS cancer patients are generally mild, even subtle changes in cognitive function can have a significant impact on a patient’s quality of life [9].

About 50% of the 3.45 million people diagnosed with cancer each year in Europe is part of the working population [10], of whom 89% manage to return to work (RTW) within 24 months [11]. Cancer patients rate employment as the third most important aspect of their quality of life [12]. Being able to work is a sign of recovery, and it contributes to patients’ self-esteem. Still, cancer survivors report effects of cognitive problems, such as problems related to memory, concentration, attention, and executive functioning, as highly affecting their work performance [11, 13–15]. Avoidable work disability and consequent productivity losses should be reduced. From both a patient and a societal perspective, it is therefore of relevance to provide evidence-based treatment options for working non-CNS cancer survivors confronted with persistent cognitive problems.

Whereas numerous studies have been performed evaluating the effectiveness of interventions to support cancer patients in their RTW process [16], there is a lack of interventions to accomplish sustainable employability [17]. From the literature so far, cognitive impairment seems to be only weakly associated with RTW [13], but its impact on work-related problems at the workplace appears to be substantial [10]. This underlines the need for effective vocational cognitive rehabilitation, implemented in a phase that patients already have achieved RTW.

So far, evidence-based treatment options for cancer survivors experiencing cognitive problems are limited [18, 19]. In other neurological patient populations, meta-cognitive strategy training has shown to be successful in improving daily life functioning, using intact cognitive abilities and strategies together with psycho-education. By teaching patients compensatory strategies to achieve the same behavior in a different way, intact neural circuits might be reorganized and different neuropsychological systems might be used [20]. Meta-cognitive strategy training appears to be a promising treatment for cancer patients as well. That is, there is evidence regarding improvement in areas of daily life functioning, in self-perceived cognitive function, and to a lesser extent, in tested cognitive function [6, 18, 21–24]. However, since results are neither robust nor consistent, additional studies are required to establish effectiveness of meta-cognitive strategy training for non-CNS cancer survivors.

Internet-based interventions are gaining ground in the field of cognitive rehabilitation [25–30] as they empower self-management regarding patients’ own health care. Cancer survivors perceive flexibility in use (i.e., accessible where and when the patient likes) as an important component to the development of any treatment for cognitive changes [31]. Furthermore, Internet-based delivery may be more economical than face-to-face therapy as it requires less therapist time. In addition, the overall mean effect size of Internet-based psychotherapeutic interventions is comparable to the effect size of traditional face-to-face interventions, indicating that these interventions are an effective treatment method [32].

In the current study, we will develop both a basic and an extensive version of an Internet-based cognitive rehabilitation program for occupationally active cancer survivors with persistent cognitive problems. The intervention consists of different elements from meta-cognitive strategy training that have been shown to be effective in managing symptoms, such as problems related to memory, concentration, attention and executive functioning, and fatigue. In this article, we describe the design of a randomized, controlled, multicenter trial in which the (cost-)effectiveness of this Internet-based cognitive rehabilitation program on work-related outcomes will be evaluated. It is hypothesized that both cancer survivors who undergo the basic or extensive cognitive rehabilitation program will achieve more often their pre-set work-related rehabilitation goals, compared to cancer survivors in the waitlist control group. Furthermore, it is hypothesized that cancer survivors with cognitive impairment at study entry (as measured with neuropsychological tests) will be better at achieving their pre-set rehabilitation goals when allocated to the extensive cognitive rehabilitation program compared to the basic cognitive rehabilitation program. Finally, we hypothesize that both the basic and extensive cognitive rehabilitation program will be cost-effective compared to the control group. If found to be (cost-)effective, this Internet-based cognitive rehabilitation program can be embedded as standard practice in the growing community of occupationally active cancer survivors experiencing cognitive problems at work.

## Methods/design

### Study design

A multicenter, three-armed randomized controlled trial will be conducted to evaluate the effectiveness of an online cognitive rehabilitation program. Two intervention groups (i.e., a basic and an extensive cognitive rehabilitation program) and one waitlist control group will be included. An economic evaluation will be conducted to assess cost-effectiveness of the cognitive rehabilitation program. Furthermore, a process evaluation will be performed to evaluate the procedures regarding recruitment, execution and implementation of the cognitive rehabilitation program. This study will be conducted by the Netherlands Cancer Institute, Amsterdam, the Netherlands. Participants will be cancer survivors who are treated for cancer in several (university) hospitals in the Netherlands. The study has been approved by the Medical Ethic Committee at the Netherlands Cancer Institute and is registered at ClinicalTrials.gov (registration number NCT03900806).

### Study sample

The study sample will be composed of 261 men and women of working age (18–65 years) with histologically confirmed non-CNS cancer. Eligible participants should have been treated with chemotherapy, targeted agents, and/or immunotherapy, which should have been completed a minimum of 6 months before study entry. Patients who are still receiving hormonal therapy can be included in the trial. Since it is expected that cognitive problems affect work in the first period after RTW most prominently, cancer diagnosis can be at the utmost 42 months ago at study entry. Eligible patients should self-report cancer and/or cancer treatment-related cognitive problems. This will be assessed by the study team during a screening. Both patients with and without cognitive impairment according to neuropsychological tests (i.e., a *z*-score of 1 under or above the mean score of a control group, on at least two tests of the different cognitive domains compared to the normative data of a healthy population by age) will be included in the study. Furthermore, eligible participants should be occupationally active for a minimum of 12 working hours per week and have a fixed or temporary employment contract (with at least 6 months left of their contract).

Patients will be excluded from the trial if they lack basic proficiency in Dutch, have a serious overt psychiatric or neurological disorder that can interfere with the study aims, or have no Internet access. In addition, patients will be excluded if they participate in comparable studies or programs focused on the reduction of cognitive problems and/or on the support of patients to retain work.

### Recruitment and randomization

Cancer patients will be identified through the Netherlands Cancer Registry and recruited via treating physicians of several (university) hospitals in the Netherlands. Potentially eligible patients will receive a personalized letter from their treating physician, informing them about the study and the cognitive rehabilitation program. Patients will be asked to respond via post (response card), email, or phone whether or not they are interested in participation. In case a patient does not respond, a reminding letter will be sent after 3 weeks. Patients who express interest in participation will receive a phone call from the study team for further screening (e.g., a check of their employment status) and to provide additional information about the trial. Patients will be invited by e-mail to complete the baseline measurement (i.e., an online questionnaire and an online neuropsychological assessment) in case they fulfill all criteria, are motivated, and are willing to comply to the trial procedures. After completion of this baseline measurement, a session (face-to-face or using videoconference) with a therapist (e.g., neuropsychologist, occupational therapist) will be planned to discuss outcomes of the neuropsychological assessment, followed by collaborative treatment goal setting.

**Fig. 1:**
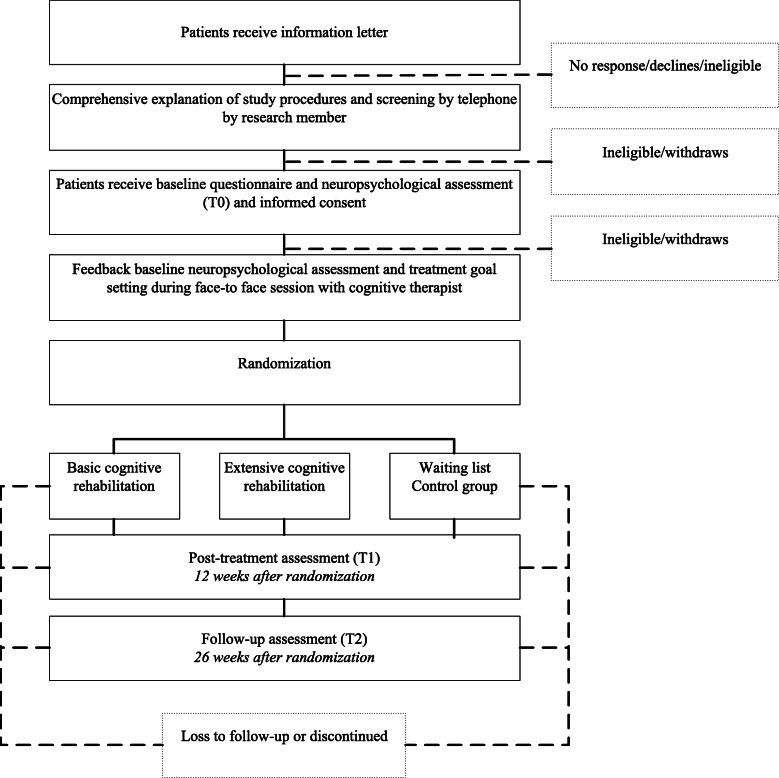
Overview of the study design

**Fig. 2:**
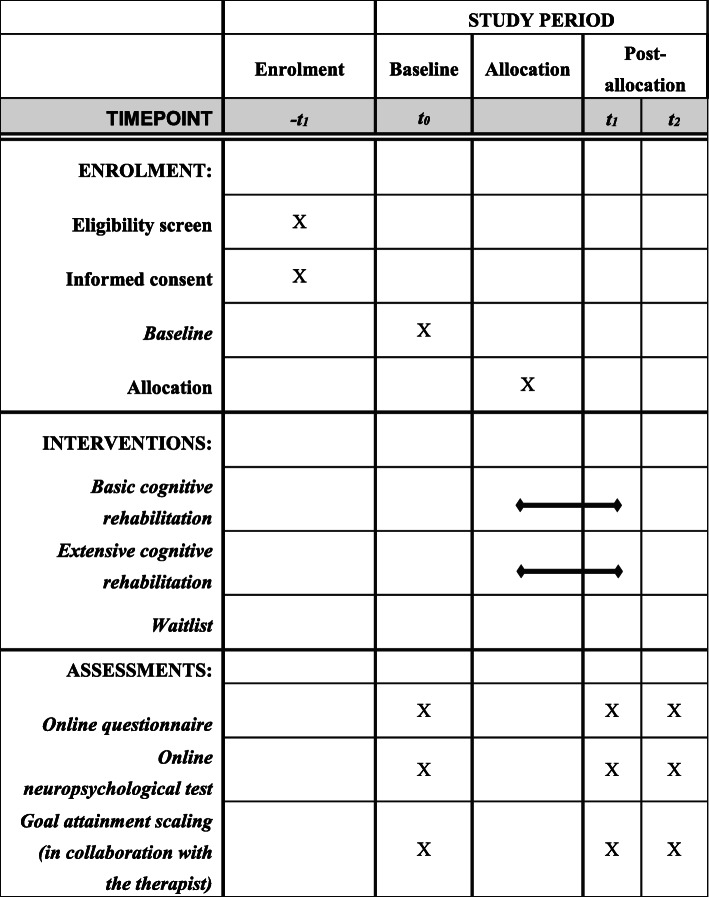
Schedule of enrolment, interventions, and assessments

Upon receipt of all baseline information and after the session with the therapist, patients will be randomly allocated to the basic intervention arm (*N* = 87), the extensive intervention arm (*N* = 87), or the waitlist control group (*N* = 87), using randomized design in the computerized program ALEA. Minimization will be applied for the presence of cognitive impairment on the neuropsychological assessment to equalize group sizes. Blinding of participants and professionals is not possible for this type of intervention. Further, blinding for assessment is not applicable since measurements will be computer-based. A flow diagram of the recruitment procedure is outlined in Fig. 1. Figure 2 shows a schedule of enrolment, interventions, and assessments.

### Intervention

The cognitive rehabilitation program will be based on the protocol of a frequently used meta-cognitive strategy approach applied in many rehabilitation centers in the Netherlands (i.e., “Niet Rennen Maar Plannen”). In cooperation with the creators of this protocol, we will adapt it for work-related cognitive problems and for online use. Patients and professionals will evaluate the adapted version of the protocol in several stages throughout its development. Both individual sessions and focus group interviews will be organized to obtain feedback, which will be used to evaluate and improve both content and user-friendliness of the adapted version of the intervention. The program will consist of several modules that can be used in a flexible way, depending on the specific individual problems and goals. As part of this study, therapists have undergone additional training in issues related to cancer-related cognitive impairment and problems occupationally active cancer survivors might experience at work. In both intervention arms, participants will receive access to a secured personal webpage, where all content relevant to the treatment sessions can be obtained. The basic and extensive intervention arms differ with regard to (1) therapy guidance (i.e., absent in the basic arm and present throughout the whole program in the extensive arm) and (2) intervention content. Table 1 shows an outline of the intervention content of the basic and the extensive cognitive rehabilitation program. The number and duration of sessions of both intervention arms may vary according to the individual needs and preferences of the participants, in line with the original face-to-face program on which the interventions are build. Therefore, there is no fixed number or duration of sessions in both intervention arms.

**Table 1:** Outline intervention content

Modules	Content	Basic cognitive rehabilitation	Extensive cognitive rehabilitation
1. Psychoeducation	▪ Review current knowledge about cancer related cognitive impairment ▪ Learning how to identify “at risk” situations (at work) where cognitive failures arise	X X	X X
2. Fatigue-management	▪ Coping with fatigue (at work)	X	X
3. Cognitive behavioral therapy	▪ Coping with behavioral and emotional consequences of cognitive impairment (at work)	X	X
4. Communication	▪ Learning how to disclose your cognitive problems to colleagues	X	X
5. Strategy training: memory	▪ Learn and practice memory strategies at the workplace		X
6. Strategy training: information processing	▪ Learn and practice information processing strategies at the workplace		X
7. Strategy training: executive function	▪ Learn and practice planning and problem solving, flexibility and self- control strategies at the workplace		X
Therapy guidance			X

#### Basic cognitive rehabilitation program

The basic arm will consist of a brief cognitive training program without individual guidance throughout the intervention, which has to be completed in a period of 12 weeks.

#### Extensive cognitive rehabilitation program

The extensive arm will consist of a comprehensive training program, which has to be completed in a period of 12 weeks. The extensive cognitive rehabilitation program involves tailored therapy guidance in which the patients’ in-session reflection and homework assignments will be discussed. Cognitive strategy modules will be assigned by the therapist depending on the participants’ cognitive profile and personal treatment goals.

#### Waiting list control group

Participants in the waiting list control group will be offered the opportunity to follow the basic cognitive rehabilitation program after completion of the 26-week follow-up measurement.

### Data collection

**Fig. 3:**
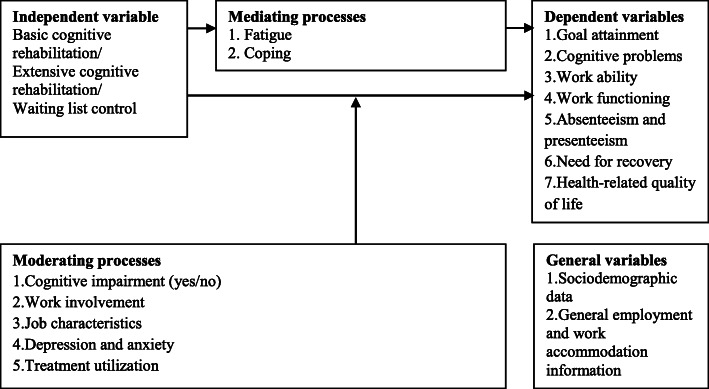
Dependent and independent measures, mediating and moderating processes

Participants will be followed for 6 months in total and will be invited to complete measurements at three time points (at baseline (T0), treatment endpoint at 12 weeks post-randomization (T1) and at 26 weeks post-baseline (T2)). Measurements will be performed via a secured website, for which a link will be sent by email. Intervention effectiveness will be measured in terms of work-related goal attainment and secondary outcome measures. Secondary analyses will be performed to explore moderating and mediating processes. An overview of measures and mediating and moderating processes in treatment is presented in Fig. 3. Detailed descriptions of these secondary study measures are provided in Table 2.

**Table 2:** Study measures and corresponding instrument

Variable	Instrument	Details
*Primary outcome*		
Goal Attainment Scaling	GAS	▪ 3 personalized treatment goals
		▪ 6-point scale
*Secondary outcomes*		
Cognitive problems	CSC-W DV	▪ 19 items, 5-point scale
		▪ Score range: 0 (never) to 4 (always)
		▪ Cronbach’s alpha: 0.95
Work ability	WAI	▪ 1 item, 10-point Likert scale
Work functioning	WRFQ	▪ 27 items
		▪ Range 0 to 100; higher scores indicate better work functioning.
		▪ Subscales: work scheduling demands, mental demands, social demands, physical demands, and output demands
		▪ Cronbach’s alpha: 0.91–0.96
Absenteeism and presenteeism	iPCQ	▪ 6 items
		▪ Subscales: absenteeism, presenteeism
Need for recovery	VBBA	▪ 11 items (subscale)
Health-Related Quality of Life	SF-36	▪ 36 items, dichotomous and 3- to 6-point Likert scales
		▪ Subscales: vitality, physical functioning, bodily pain, general health perceptions, physical role functioning, mental health, emotional role functioning, and social role functioning
		▪ Score range: 0–100; higher score indicates higher levels of functioning/ well-being.
		▪ Time frame: past week
		▪ Cronbach’s alpha: 0.66–0.91 (mean 0.84)
Fatigue	SF-36	▪ 4 items (subscale)
Coping	CERQ	▪ 36 items, 5-point Likert scale
		▪ Score range: 0 (never) to 5 (always)
		▪ Subscales: self-blame, acceptance, rumination, positive refocusing, refocus of planning, positive reappraisal, putting in to perspective, catastrophizing and other blame
Neuropsychological function	ACS	▪ 7 different neuropsychological tasks
		▪ Online assessment
		▪ Cognitive domains: executive functioning, information processing speed, attention, working memory, verbal learning and memory, psychomotor speed.
		▪ The ASC is usable a reliable for the oncology setting, with test–retest correlations in the range 0.29 up to 0.78, which is comparable with traditional tests.
		▪ Concurrent validity with traditional tests is medium to large.
Work involvement	WIS	▪ 6 items, 5-point scale
		▪ Score range: 1 (totally disagree) to 5 (totally agree). High scores indicate a high work involvement
Job characteristics	JCQ	▪ 35 items, ordinal 4-point scales
		▪ Score range: 1 (totally disagree) to 4 (totally agree).
Depression and anxiety	HADS	▪ 14 items, 4-point Likert scale
		▪ Subscales: depression, anxiety
		▪ Score range: 0–42
		▪ Time frame: past week
		▪ Cronbach’s alpha: 0.68–0.93
		▪ Cut off: a score of 11 and over indicates the possible presence of clinical depression.

### Study measures

#### Sociodemographic and clinical data

Sociodemographic data including age, gender, marital status, number of children living at home, education, breadwinner status (sole/shared) will be obtained via questionnaire. Clinical information including cancer site, month/year of diagnosis, received (and future) treatment(s), (i.e., surgery, radiation, chemotherapy, immunotherapy, targeted treatment, hormonal therapy other medication), recurrence(s) of the disease, and comorbidity will be obtained from the medical records, Netherlands Cancer Registry, and via self-report. Information on general employment issues and on work accommodation, including employment sector, type of employment (fixed/temporary), years of work experience, and working hours and days per week according to employment contract, will be acquired via questionnaire.

#### Primary outcome measure

The primary outcome for this study will be individually defined work-related treatment goals, using the 6-point Goal Attainment Scale (GAS) on personal outcome (− 3, achievement of the goal after training is worse; − 2, achievement of the goal is the same; 1, partial achievement of the goal; 0, achievement of the goal; 1, exceeding the goal; and 2, greatly exceeding the goal) [33]. Patients will formulate three treatment goals and define the six outcome levels at baseline, in collaboration with their therapist. Attainment of the goals is measured in a standardized way, i.e., an overall GAS *T*-score will be computed for each participant on the basis of aggregated GAS scores involving attainment of multiple personal treatment goals, according to a summary scoring algorithm that calculates the extent to which patients’ goals are met.

$$ T=50+\frac{10\sum {w}_i{x}_i}{{\sqrt{\left(1\hbox{-} \rho \right)\kern0.5em \sum {w_i}^2+\rho \left(\sum {w}_i\right)}}^2} $$

*T* is the composite score, *w*_*i*_ is the weight assigned to goal_*i*_ (based on the patients prioritization), *x*_*i*_ is the original score for goal_*i*_ ranging from − 3 to + 2, and *ρ* is the estimated correlation between goal scores. The *T*-score has a mean of 50 and a standard deviation of 10. *w*_*i*_ is considered as equally relevant for each goal and thus assigned 1. Based on previous studies, correlation between the goal scores is constant and can be set at 0.3 [33]. Goals will be set at baseline (T0), and evaluated by patients (of all study arms) in collaboration with the cognitive therapist at T1 and T2. Due to practical reasons, this scoring of the level of attainment will be done by telephone. An independent assessor will perform quality checks on the use and scoring of GAS during the initial phase of the trial, to assure fidelity of the GAS-protocol.

#### Secondary outcome measures

Secondary outcome measures include standardized self-report questionnaires assessing cognitive complaints (CSC-W DV) [34], work ability (WAI) [35, 36], work functioning (WRFQ) [37], absenteeism and presenteeism (iPCQ) [38], need for recovery after a working day (VVBA) [39, 40], and health-related quality of life (SF-36) [41].

#### Mediation and moderating measures

Fatigue (SF-36) and coping (CERQ) are considered mediators and will be assessed through self-report questionnaires [42, 43]. Neuropsychological functioning is considered a moderator and will be measured using a self-administered online neuropsychological test battery (ACS) [44]. Other moderators are work involvement (WIS) [45], job characteristics (JCQ) [46], depression and anxiety (HADS) [47], and treatment utilization (Log-data of the online program).

### Cost-effectiveness

An economic evaluation will be conducted alongside the trial to evaluate costs and patient outcomes of implementing the online cognitive rehabilitation program. Both cost-utility analysis (CUA) and cost-effectiveness analysis (CEA) will be conducted. Quality-adjusted life years (QALYs) will be measured using EQ-5D-5L [48]. Intervention costs will be calculated, including health professional labor (time spent on treatment per patient), staff training, administration, and material costs. Unit prices (or appropriate tariffs) will be multiplied by volumes of use, following the Dutch costing guidelines [49]. Health care costs will be assessed using self-reported questionnaires about participants’ use of health care services (e.g., general practitioner (GP), medical specialist, paramedical care). Productivity costs will be administered by questionnaires reporting on productivity losses due to sickness absence from work (absenteeism) and health-related diminished productivity at work (presenteeism). Presenteeism and sickness absence will be administered with the Productivity Costs Questionnaire (iPCQ). This questionnaire includes three questions for measuring absenteeism and three questions identifying the proportion of presenteeism [38]. Full working days lost because of presenteeism are calculated according to following formula: *P* = (*E* − *A*)∗ *p*, in which *E* is the total working days, *A* is sickness absence days, and *p* is the proportion of lost work performance due to presenteeism. Productivity loss will be valued using age-, gender-, and/or education-specific price weights.

### Process evaluation

A process evaluation will be conducted to evaluate strategy fidelity, satisfaction, and facilitators or barriers for implementation of the cognitive rehabilitation program. Six components will be assessed, namely, recruitment, reach, dosage, differentiation, implementation, and experiences (of both participants and therapists). We define recruitment as the result of all procedures to recruit eligible cancer survivors for participation. Reach is defined as the percentage and characteristics of persons who receive the intervention program. The extent to which the participants actually were exposed to the intervention will account as treatment dosage. Furthermore, differentiation is regarded as the identification of unique and essential intervention features. Implementation refers to the qualitative aspects of the manner in which the intervention program was delivered. Participants’ and therapists’ experiences with the intervention activities will be evaluated. To gather data for the process evaluation, periodic self-report questionnaires for participants will be provided at the end of every module (for an overview of modules, see Table 1) and at T1. A self-report questionnaire for the therapists will be provided at the end of the trial.

### Sample size calculation

This longitudinal study design will allow for testing of the main effect of the intervention over time, with the GAS score as the primary outcome measure. With a sample size of 65, and an alpha = 0.05, the study will allow for an attrition rate of approximately 20% and have 80% power to detect an effect size of *f* = 0.2 (equivalent to Cohens *d* = 0.4) for the main effect of the intervention between both the basic and extended cognitive rehabilitation treatment group versus the waitlist control group (first hypothesis). To perform subgroup analysis, used to test our second hypothesis, sample size should be inflated fourfold [50]. Therefore, we strive for a sample size of 261, with 87 patients in each group.

### Statistical analysis

All data will be pseudo-anonymized prior to data analyses. The data set will not contain any personal identifiers. The information that is given online by patients is accessible to the study staff only, via a secured code. Means and SDs will be presented for continuous, normally distributed variables, and median and ranges for non-normally distributed variables. First, analyses will be performed to evaluate the comparability of the three study arms (i.e., the basic and extensive cognitive rehabilitation groups and the waiting list control group) at study entry, in terms of sociodemographic and clinical characteristics. ANOVA tests or appropriate non-parametric statistics will be used, depending on the level of measurement. If, despite the stratified block randomization procedure, the groups are found not to be comparable on one or more background variables, then those variables will be employed as covariates in subsequent analyses.

In this study, GAS will be used as the primary study endpoint, evaluating between-group differences over time in GAS scores. The GAS scores will be calculated according to published scoring algorithms (see the “Methods/design” section). To evaluate intra-individual differences in the trajectory of change over time for the primary outcome, we will use a growth curve modeling approach with random intercept and slope. This approach takes into account the within- and between-person variability and deals adequately with missing data [51]. Both linear and quadratic effect of time will be modeled to determine if an initial improvement or deterioration in the outcome was followed by a deceleration of this change over time. Appropriateness of the final model (with or without quadratic effect) will be determined based on model fit statistics: the Bayesian information criterion [52] and the Akaike information criterion [53]. In all analyses, the control group will be the reference group. Furthermore, moderation analysis will be conducted to determine whether the interventions have a differential effect among the subgroups classified as cognitive impaired yes or no (measured by ACS) at baseline. Baseline differences will be accounted for in the model. In case of non-ignorable dropout, we will correct the model for different patterns of missing values [54]. All analyses will be done on an intention-to-treat basis. Furthermore, as a secondary analysis, per-protocol analyses will be performed on patients who met criteria for minimal compliance (i.e., at least 4 logins into the online program) with the intervention(s). Differences in mean change scores over time between the intervention groups and the control group will be accompanied by effect sizes. Effect sizes of 0.2 are considered small, 0.5 moderate, and 0.8 large [55]. Effect sizes of approximately 0.5 are considered to be clinically relevant [56].

#### Secondary study parameter(s)

We will calculate questionnaire scores according to published scoring algorithms. Missing values will be replaced by the average score of the completed items in the same scale for each participant, provided that at least 50% of the items in that scale have been completed. For the CSC-W DV, WAI, WRFQ, iPCQ, VBBA, and SF-36, the same growth curve modeling approach as described in the primary study parameter section will be used to evaluate between-group differences over time. In addition, we will test whether intervention effects are moderated by baseline scores on the ACS, WIS, JCQ, and HADS. Finally, we will explore the mediating effect of the (anticipated time-variant) variables fatigue (SF-36) and coping (CERQ) on the secondary outcomes.

### Cost-effectiveness

Incremental cost will be calculated and, by using incremental QALYs and GAS scores, expressed as the incremental cost-utility ratio (ICUR) and incremental cost-effectiveness ratio (ICER), respectively. The ICUR and ICER will be measured for both intervention groups. ICUR represents the costs required for the particular intervention to generate one additional QALY in comparison with care as usual (control group). ICER represents incremental costs per relevant achievement of work-related treatment goals (GAS) over a time period of 6 months. Both ratios will be estimated by bootstrap analyses. As an indication of whether an intervention will be considered cost-effective, the ICUR is compared to a range of ceiling ratios varying from €20k per QALY to €80k per QALY in the Netherlands, with €30k per QALY commonly accepted as the prevailing ceiling ratio. In addition, cost-effectiveness acceptability curves will be used to inform decision-makers on the probability that the cognitive rehabilitation program is cost-effective.

## Discussion

In the current study, we will evaluate the (cost-)effectiveness of both a basic and an extensive version of an online cognitive rehabilitation program on work-related goal attainment, subjective cognitive functioning, work ability, work functioning, absenteeism and presenteeism, need for recovery, and quality of life.

Our study has the following notable strengths: (1) the randomized trial design, (2) the multicenter nature of the trial, (3) the comparison of two intervention groups with a waiting list control group, (4) the selection of patients based on the presence of cognitive problems, (5) the use of a personalized treatment outcome (GAS) to capture meaningful work-related outcomes; and (6) the inclusion of a cost-effectiveness analysis and process evaluation.

Despite these strengths, some shortcomings should be taken into account. First, the basic version of the cognitive rehabilitation program differs from the extensive version in two respects, namely, content and therapy guidance. It might be the case that one of the versions is more or less effective than the other. If so, distinguishing between the efficacy of those two respects is not possible. However, we have chosen this design with this limitation, as it gives us the opportunity to examine if a low intensive, parsimonious approach that can be easily implemented and is inexpensive might be sufficient to treat cognitive problems in occupationally active cancer survivors, or that a more extensive approach is needed for (a subgroup of) patients. Second, although the use of the personalized treatment outcome (GAS) is a major strength, it comes with disadvantages as well. Two of those issues are (1) its lack of a clear guide in how to interpret the aggregated GAS *T*-score and (2) challenges in defining realistic and solid GAS scales. To overcome the first issue, GAS scores will be accompanied by effect sizes so that it will be appropriate to draw conclusions about effectiveness. With regard to the second issue, we will train the involved therapists in the use of GAS. Besides, an independent assessor will perform quality checks on the use and scoring of GAS during the initial phase of the trial.

To conclude, given the adverse impact of cognitive problems on a cancer patient’s work performance, there is need for effective and accessible treatment options. If proven to be (cost-)effective, our Internet-based cognitive rehabilitation program will be a valuable addition to standard care for the growing community of cancer survivors experiencing cognitive problems at work.

## Data Availability

Not applicable.

## Abbreviations

CNS: Central nervous system
CRCP: Cancer-related cognitive problems
CUA: Cost utility analysis
CEA: Cost-effectiveness analysis
QALY: Quality-adjusted life years
ICUR: Incremental cost utility ratio
ICER: Incremental cost-effectiveness ratio
